# The potential of single session intervention approaches to enhance the mental health and resilience of older adults, care partners, and healthcare systems

**DOI:** 10.3389/fpubh.2025.1515440

**Published:** 2025-03-05

**Authors:** Sarah M. Bannon, Andy Rapoport, Allison J. Applebaum, Jessica L. Schleider

**Affiliations:** ^1^Department of Rehabilitation and Human Performance, Icahn School of Medicine at Mount Sinai, New York, NY, United States; ^2^Department of medical social sciences, Northwestern University, Evanston, IL, United States; ^3^Memorial Sloan Kettering Cancer Center, New York, NY, United States

**Keywords:** single session, brief intervention, older adults, care partners, caregivers, mental health

## Abstract

Single-session interventions (SSIs) are mental health (MH) interventions that intentionally involve a single encounter. In this commentary, we outline issues with existing models of MH care for older adults & their care partners, how SSIs can address barriers, and considerations for research. We encourage the development of SSIs to increase accessibility, scalability, participation, and cost-effectiveness of mental health interventions.

## 1 Introduction

The mental healthcare needs of older adults and their care partners (i.e., family members and other close affiliates providing unpaid care) are rapidly increasing with the aging population. By 2030, one in six people globally will be aged 60 years or older, and by 2050, this demographic will double to 2.1 billion (1). Older adults and their care partners report high rates of clinically elevated emotional distress, significant unmet psychosocial support needs, and dissatisfaction with many aspects of their care.

Addressing the mental health needs of older adults and care partners is infeasible with predominant intervention models. Specifically, standard psychotherapy programs are time-intensive (typically 10–12 sessions), delivered by professionals with advanced training, and available in limited contexts. Mental healthcare remains disorder-driven or focused on single conditions (vs. strengths-driven or accounting for co-occurring conditions). This is a poor fit for older adults and care partners, who frequently navigate comorbid conditions and are interested in strengths-based approaches (2, 3). Recently, evidence-based interventions have been developed for older adults and care partners that are transdiagnostic (i.e., not specific to disease or condition) in nature (4–6). However, most are not feasible to implement at a large-scale due to the time, resources, and trained workforce required.

There is an urgent need for more scalable and sustainable avenues to address the mental healthcare needs of older adults and their care partners. Single-session interventions (SSIs) may represent one possible avenue. SSIs are defined as specific brief and low intensity interventions that intentionally involve a single in-person or online visit or encounter with a program, provider, or service (7). SSIs serve to make treatment more widely available, meeting people where they seek care, and they offer an approach suitable for those who would not otherwise engage in longer-duration interventions. SSIs can be self-guided or facilitated by a professional or lay provider; they can be digital or conducted in-person. By offering support rapidly and in a low-intensity format, SSIs have demonstrated success among high-need populations that may otherwise receive no treatment, such as those on waitlists for mental health clinics (8–11).

Despite their potential benefits, there are few established SSIs specifically designed to address the needs of older adults and their care partners. This commentary highlights the potential of implementing SSIs for older adults and their care partners. Below, we outline (1) unmet needs and issues with existing models of care, (2) potential intervention mechanisms, targets, and benefits of SSIs, and (3) considerations for future research. We aim to encourage the development of SSIs within and outside of healthcare settings to potentially increase accessibility, scalability, participation, and cost-effectiveness of mental health interventions to support older adults and care partners.

## 2 Unmet needs and issues with existing models of care

Advances in healthcare and improvements in living conditions have successfully prolonged lifespans, but at the same time have led to an increased likelihood for chronic illness exposure and age-related health concerns (12). Older adults experience high rates of co-occurring chronic health conditions (e.g., chronic pain, arthritis, cardiovascular disease, diabetes, depression, and dementia), aging-related impairments of multiple organ system (e.g., frailty, falls, incontinence, delirium), and resultant disability that are “rules rather than exceptions” (1, 13). These issues are emotionally taxing and difficult to navigate for individuals and their care partners who are often described as the “invisible” patients or workforce. Over one-third older adults and their care partners endorse high rates of emotional distress (14, 15). Among older adults—and particularly those with chronic conditions—emotional distress is associated with increased healthcare usage, which places a toll on healthcare systems (16, 17).

Among older adults and care partners, several important structural barriers to mental healthcare exist, including:

### 2.1 Growing demands and insufficient trained workforce

Prior generations of adults ages 65 and older were less likely to report concerns to any providers other than primary care physicians, and were less likely to access mental healthcare relative to younger adults (18). However, younger generations including the “baby boomers” that reached >65 in 2020, demonstrate worse mental health than older generations and a greater likelihood to seek mental healthcare services (19). In the decades ahead, there will be a growing demand for mental healthcare. This is an important issue given the lack of appropriately trained professionals (e.g., psychologists, counselors, social workers) to provide services for older adults across cares settings (20). When longer-duration mental health programs are developed, they are often not sustainable to implement in healthcare settings due to the time demands placed on providers (21).

The lack of available workforce is even more of a barrier for care partners, who suffer from significant morbidity themselves and are not considered in routine assessment and mental health support (22). In recent years, U.S. policy changes such as the state-level Care, Advise, Record, Enable (CARE) Act (23) and the 2023 Center for Medicare and Medicaid Innovation (CMMI) Guiding an Improved Dementia Experience (GUIDE) model (24) have encouraged the inclusion of family care partners in comprehensive care packages focused on care transition, coordination, and respite services. While these and other policy initiatives have led to some system-level changes, scheduling difficulties and poor efficiency of care delivery are areas requiring improvement (23, 25).

### 2.2 Long wait-times for outpatient mental healthcare

The demand for mental healthcare dramatically exceeds the number of available providers, leading to long waits being the norm. In the U.S., wait-times for outpatient psychotherapy can last upwards of weeks, even several months (10, 26). Longer wait-times contribute to worsening mental health symptoms (27), even when compared to “no treatment” conditions, in which individuals are not expecting future care (10, 28). Long wait times lead to smaller symptom improvements and increased likelihood of early dropout once mental healthcare has begun (10, 29, 30). Waitlists and delays from referral to visit time in outpatient mental health clinics lead to worse outcomes (e.g., pain, function, physical activity, and mortality), particularly for individuals experiencing frailty, undergoing evaluation for transplants, and following cardiac events (31, 32).

### 2.3 Time and effort to engage in mental healthcare

Both older adults and care partners face barriers to engaging in typical duration mental healthcare due to time commitment expectations. Approximately 42% of older adults do not return to mental health interventions after the first session, which increases to 75% after 6 months of treatment (33). Older adults cite a lack of early relief in symptoms as the top reason for dropout (33), suggesting the need for more potent intervention approaches. Older adults often prefer to seek help from their primary care providers for mental health concerns, and providers cite lack of time as an important barrier to intervention (34). Among care partners, dropout from mental health interventions is high (~50%) (35, 36), and many refuse to engage in interventions altogether due to the required time commitments (37).

### 2.4 Lack of embedded mental healthcare for changes in cognition and increased dependency

Many older adults experience changes in memory and cognition, and report anxiety surrounding aging. Performance on cognitive assessment is highly sensitive to individuals’ stress surrounding testing (38). Early mental healthcare is seldom available for older adults experiencing progressive neurocognitive symptoms, leaving many without support during the “window of opportunity” where they can meaningfully participate in interventions (39).

### 2.5 Absence of mental health support for acute medical events and care transitions

Older adults and care partners are also increasingly present in emergency and critical care settings, and note a lack of mental health support (40). Older patients experience more frequent care transitions from hospital to home and across facilities relative to younger patients given their increased experience of co-occurring conditions, cognitive impairment, and functional status (41). Care transitions are often challenging times, and rehospitalization is common (41, 42). Specific challenges include: missing information regarding diagnoses and symptoms, a lack of follow up care, new physical health concerns that complicate transition plans, care partner distress, and disagreements on how to navigate care (42). Care transitions will continue to increase as the population ages, leading to a growing interest in “rapid rehabilitation” (43). Acute and transitional care models for discharge that improve the mental health of patients and care partners exist, though most are inaccessible or not feasible to implement in large scale contexts due to time, cost, geographic, and workforce resource constraints (44).

## 3 Potential benefits, targets, and mechanisms of SSIS

SSIs may be more equipped to address the challenges related to growing demands for mental healthcare support for older adults and care partners in clinic and community settings relative to longer duration interventions (9). SSIs are optimized to achieve meaningful clinical progress within just one session, though visits are sometimes added as needed (i.e., in a “one-at-a-time” manner). This approach may offer specific benefits for participants that are unlikely to attend beyond the first session, which is a substantial portion of older adults and care partners (37). SSIs can meaningfully impact intervention targets through education, skills practice, and support that increases participants’ sense of agency, autonomy, competence, and relatedness (45, 46). A key benefit of SSIs are their *flexibility* in their format of delivery—they can be delivered as self-guided, digital interventions or implemented by trained professionals or even lay providers within existing systems and settings (e.g., healthcare clinics) (47). Having options for delivery is important given that older adults vary in their technology literacy, and may face time and geographic barriers to engaging in mental health interventions (48).

Systematic and meta reviews of the effectiveness of SSIs have shown evidence of their broad utility in improving outcomes for participants (49). However, at present there is very limited research surrounding the specific applications of SSIs for older adults and care partners. Some SSIs have been developed for adults that successfully improved a range of clinical outcomes for common challenges experienced by older adults and their care partners, including those targeting patient and care partner distress following cancer treatment, pain intensity for individuals with chronic pain, and alcohol consumption for those with frequent overconsumption (50–52).

While there is limited research on SSIs specifically developed for older adults, brief interventions (3–12 sessions) have been developed for older adults to address common challenges that can be adapted to single session formats. For example, behavioral activation is a well-documented approach to reduce depression symptoms, with some interventions demonstrating improvements in 1–2 sessions (53). In addition, compassion-based interventions demonstrate significant effects in reducing emotional distress for both older adults and care partners, though attrition is high (~18%) across studies (2, 54). Such approaches can be used to address the common challenges that older adults face surrounding shame with aging (55), and SSIs may provide an avenue to reduce attrition. Brief interventions have been developed that provide information, practical assistance, and non-judgmental support after sudden loss (56, 57) and to facilitate advanced care planning conversations among families in emergency medicine settings (58). SSIs may be used to reduce the wait times for this form of support and increase its availability. Finally, brief interventions delivered digitally to care partners of adults with chronic health conditions successfully improve care partner psychological health, self-efficacy, caregiving effectiveness, and perceived social support (59). SSIs can further facilitate care partners’ engagement by bypassing time and transportation barriers to reduce burden and improve mental health (60).

For additional SSIs to be successfully adapted, developed, tested, and implemented to address the needs of older adults and their care partners, intervention development and implementation frameworks must be considered. Specifically, the NIH Stage Model for Behavioral Intervention Development can be used to determine whether sufficient information is available on the intervention context, targets, and mechanisms to guide intervention development (NIH Stage Model Stage 0) prior to testing feasibility (61). It is important to conduct preliminary testing to identify and refine any specific modifications or adaptations that must be made to existing SSIs or longer interventions to meet the needs of older adults and care partners, and whether such approaches demonstrate early feasibility (NIH Stage Model Stages 1a-1b). Intervention targets can be selected based on what is known about the unmet needs of older adults, including emotional distress, loneliness, treatment engagement, and illness management. Mechanisms of intervention can include those hypothesized to influence attitude and behavior change in a short time frame, including self-efficacy, hope and optimism, readiness for caregiving, and growth-mindset (45, 46).

**Figure 1:**
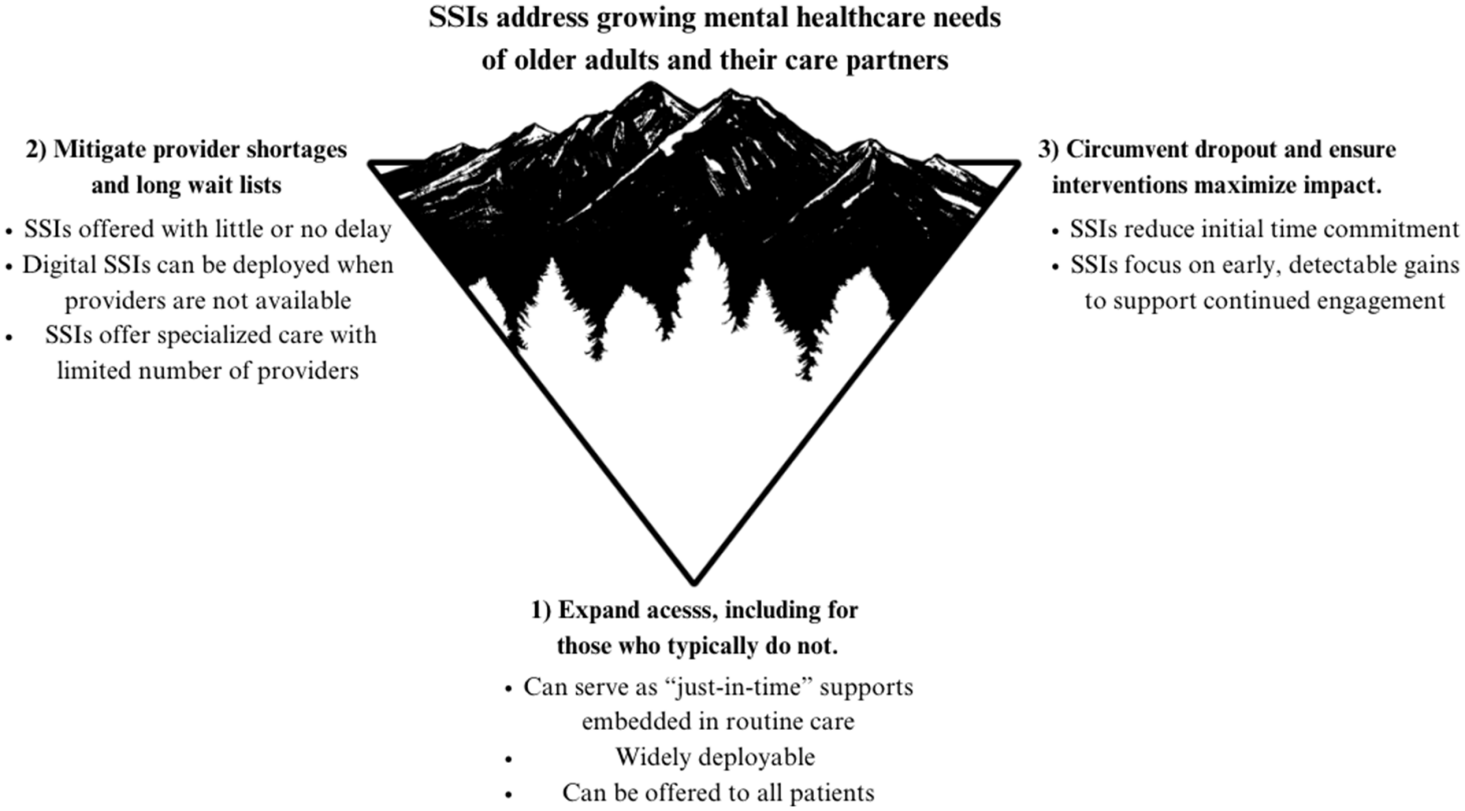
The potential of SSIs to address current gaps in care.

Ultimately, we propose that SSIs can provide avenues to increase mental healthcare support: (1) for people who never seek or access specialty services, (2) for treatment settings with provider shortages and long waitlists, and (3) to promote initial and ongoing engagement in care. Figure 1 depicts the ways in which SSIs could be used to enhance the availability of mental healthcare across treatment contexts. Below, we provide several specific examples of ways that SSIs can address gaps and barriers in care. Our goal is to encourage additional research that incorporates older adult and care partner perspectives to develop, adapt, and refine SSIs to address common challenges and increase scalable supports.

### 3.1 SSIs can expand access to mental healthcare, including to those who do not typically seek or access services

SSIs can serve as embedded, “just-in-time supports” when mental health needs arise in the context of routine healthcare settings and non-specialty clinics (e.g., with respect to emergent medical needs, cognitive decline, acute carer distress). Large scale randomized controlled trials highlight the benefits of systemwide programs for reducing depression symptoms among older adults (62). SSIs can assist in this effort, and reviews demonstrate that most users of scheduled or walk-in SSIs for common challenges in community settings (e.g., depression, anxiety, confidence) found them sufficient and helpful (63). SSIs can also be used to enhance the resiliency of communities of older adults, regardless of whether they are currently experiencing mental or physical illness (64). SSIs can be used to promote the health of entire family units and integrated into primary care clinics and community settings (e.g., public libraries, transportation, religious communities) (65). Given that older adults and care partners experience distress at high rates (66, 67), SSIs can be developed that provide coping skills for participants to effectively prevent chronic distress. While beneficial as standalone care, SSIs can also be a first intervention in a stepped care model (68), as they can be used to supplement and enhance routine assessment, bridge gaps in condition management, and delivered in either an “opt-out” or “opt-in” manner.

### 3.2 Among those interested in specialty outpatient services, SSIs can mitigate problems of provider shortages and long waitlists

SSIs can help mitigate the problems of provider shortages and long waitlists by (1) offering people help immediately when then first reach out for care as a stop-gap, (2) offering brief, evidence-based, digital support even when human-delivered care is not available, and (3) creating opportunities for non-professionals to deliver evidence-based SSIs, again circumventing the issue of limited numbers of licensed professionals.

#### 3.2.1 Outpatient clinic waitlists

SSIs can provide waitlisted patients with an interim, low-intensity support that can help mitigate adverse effects of awaiting more sustained care (10, 11). Specifically, single session consultations delivered by clinicians or lay providers have significantly decreased hopelessness, anxiety, and distress and to improve agency among patients a mental healthcare waitlist. Single session consultations have been delivered with fidelity by both trained professionals and lay providers (10, 11).

Therefore, we propose testing SSIs for waitlisted older adults patients and their care partners, focused on managing emotional distress and promoting self-efficacy. Digital SSIs can be leveraged by healthcare systems to streamline the widespread availability of support and information. For example, SSIs can be offered to all patients and care partners in an opt-in model via links sent when appointments are made to provide education and assessment of ongoing challenges. In addition, healthcare systems can also offer a provider-delivered single session consultations to waitlisted patients and care partners to begin to connect them to supports and develop coping plans. Together, these approaches can widely expand the available support and offer a flexible array of programs to participate in based on comfort with technology and preferences for in-person visits.

#### 3.2.2 Assessment and disclosure visits

SSIs can be used to help people feel more comfortable and willing to engage in testing by providing information surrounding common worries and ways of coping. For example, diagnostic disclosure visits for neurodegenerative diseases like Alzheimer’s dementia are often a stressful event for both individuals and care partners, and disclosures are linked to increased emotional distress and changes in social engagement (69). SSIs can target common challenges early after diagnosis (e.g., stigma, overwhelming emotions, reduced self-efficacy), and can offered at the time of a disclosure visit. SSIs could also be used to supplement referrals to additional supports and serve as a rapidly available intervention after screening or testing positive for a condition.

Clinics providing feedback for neuropsychological, genetic, or other clinical biomarker assessments can consider offering a digital or in-person SSI focused on coping with emotional reactions to test results and offering education on adaptive coping behaviors to adjust to changes in dependency. SSIs can also be used to link patients and care partners to additional longer-duration supports and mental health resources. Specifically, dementia care consultation models have successfully linked family caregivers to community supports after diagnosis in primary care settings (70). Such approaches could be expanded to other conditions and/or delivered at specific disclosure visits in a diagnostic process.

#### 3.2.3 Acute medical events and early distress

SSIs have demonstrated benefits for reducing depression and anxiety symptoms in older adults and care partners navigating a number of common chronic or progressive conditions (e.g., chronic obstructive pulmonary disease; dementia; mood symptoms; cognitive decline) (71, 72). Specifically, one 2 h group interventions providing cognitive behavioral therapy significantly reduced emotional distress for COPD patients relative to education only (71). Among youth patients, SSIs have demonstrated large effects on mood and behavioral concerns (73). Following acute medical events (e.g., stroke, cardiac events, falls), older adults and care partners experience myriad stressors surrounding recovery, long-term care, and end of life considerations (3). Brief individual and dyadic interventions have been developed that address emotional distress early after acute and progressive medical events (4, 74, 75), and can be adapted to focus on the most potent intervention components for digital or person-led SSIs, which can then be carefully tested and deployed in this newer population.

SSIs could be delivered during periods after acute medical events or early after diagnosis of mental or physical health conditions to reduce emotional distress, prevent risk of complications or adverse events, and to promote resiliency. SSIs exist for stroke education and slip training following falls that have improved care partner preparedness, reduce risk, and prevent and subsequent injuries (76, 77).

#### 3.2.4 Care transitions

SSIs could assist with this growing challenge by providing a context to assess and identify support needs and promote treatment engagement for all partners in care. SSIs can be developed to prioritize specific points of care transitions (e.g., prior to returning home from hospitalization; following a transition to a skilled nursing facility), to offer a context for assessing and supporting patient and care partners’ needs. Such approaches could include a digital SSI for care partners featuring education on common challenges in care transitions, and training in coping skills to manage distress and promote positive adjustment. Following a transition, SSIs could be offered to assess any issues arising during the care transition and to support coordinated care across care teams.

### 3.3 Among those who engage in mental healthcare, SSIs can circumvent dropout issues and ensure that interventions have as much of an impact as possible

SSIs reduce the time and effort to engage in care and could lower rates of dropout by increasing odds of early, detectable treatment gains. SSIs may expand the reach of mental health interventions to those who decline to participate in interventions. By focusing on the most potent content and skills, SSIs may also be able to enhance engagement in care for longer-duration interventions and management of physical conditions. SSIs could also be used to rapidly engage individuals in mental health care in community settings. Given that many older adults do not seek mental health support or are primarily interested in support from primary care providers (34), digital or hybrid SSIs could be developed for older adults and care partners that target common challenges with less of a commitment requirement than traditional interventions. For example, SSIs have been developed emphasizing ongoing screening and motivational enhancement that successfully reduce substance use among community dwelling older adults and individuals in primary care settings (52, 78, 79).

## 4 Considerations for implementation of SSIS

**Figure 2:**
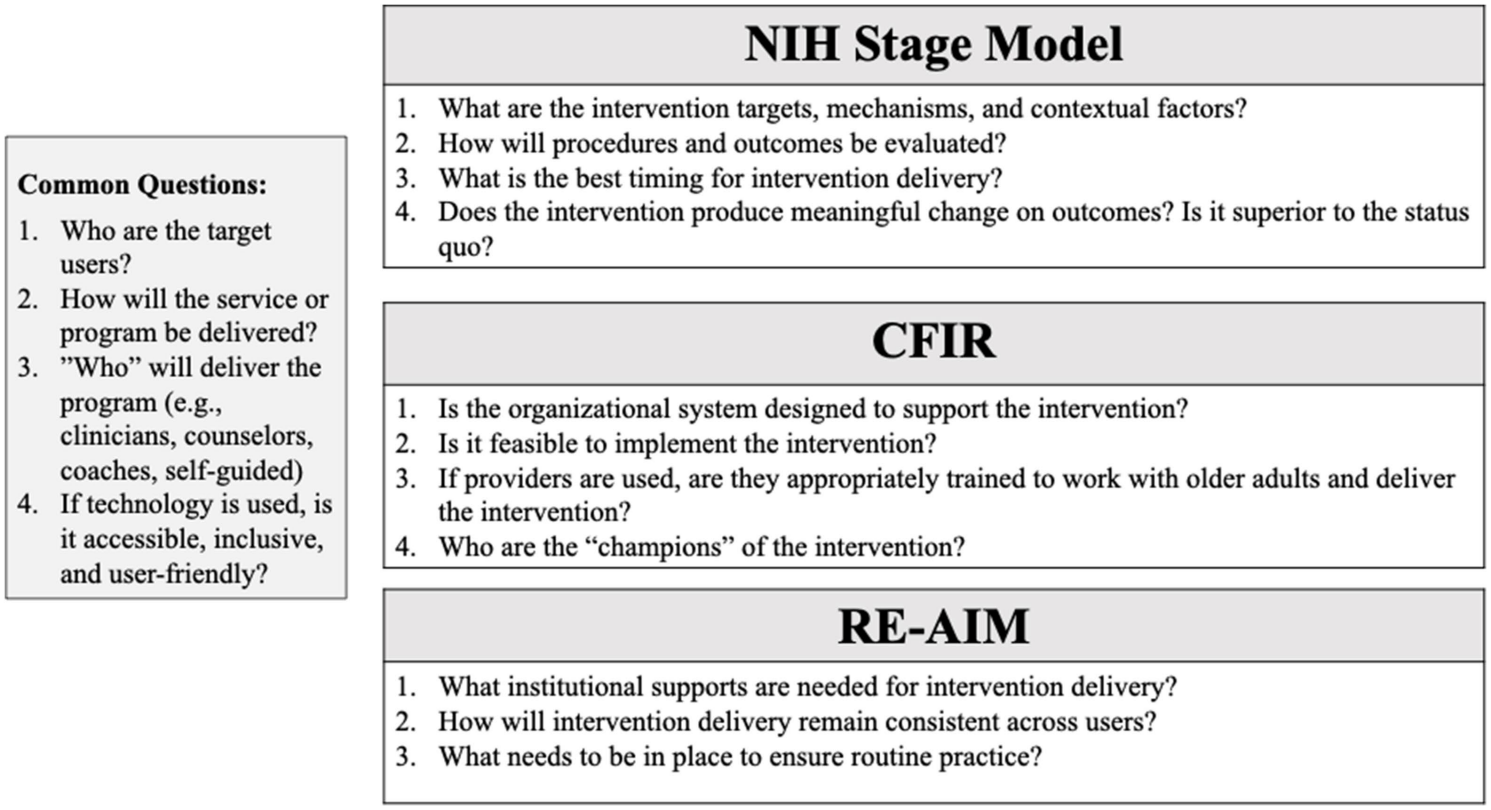
Key questions to inform the design of SSIs for older adults and care partners.

When designing new services, important questions surrounding implementation must be thoroughly considered. Implementation science frameworks such as the Consolidated Framework for Implementation Research (CFIR) (80) and the Reach Effectiveness, Adoption, Implementation, Maintenance (RE-AIM) model (81) can be consulted throughout the intervention development process to identify and evaluate factors that influence the successful implementation of interventions (82). Both the CFIR and RE-AIM frameworks consider the broader context of delivery of SSIs, and can be used to understand to best develop an intervention that fits the intended environment and target population (e.g., acute or emergency care, outpatient therapy clinics, community settings). Another key tool is the health equity implementation framework (83). This framework is designed to promote implementation equitably amongst minoritized populations. The health equity approach can be used to develop SSIs that are specifically designed to meet the needs of individuals that most consistently experience barriers in access to care, and to account for differences in needs across intended users. The goal of each of these frameworks is to better understand how factors such as intervention setting, personnel, culture, and operation might serve as barriers and facilitators to successful implementation. Figure 2 provides a description of key questions drawn from the NIH Stage Model, CFIR, and RE-AIM model that are important to consider in developing SSIs for older adults.

If SSIs incorporate mobile technology through smart devices, websites, and/or mobile applications, they should be developed with attention to the specific barriers and facilitators of technology use for older adults identified in the literature (84, 85). Mobile health interventions have demonstrated benefits on mental and physical health outcomes for older adults with chronic illnesses, though frustration with technology remain barriers to the uptake and efficacy of existing interventions (86, 87). Digital SSIs have been integrated into emails for all patients, with significant effects on aspects of resiliency such as empowerment (88). With both technology-based and in-person SSIs, interventions should be developed by meaningfully including the perspectives of the intended users.

## 5 Conclusion

Given the rapidly increasing aging population that is likely to require additional supports in the coming years, healthcare systems can begin by prioritizing the implementation of SSIs in high-need contexts (e.g., care settings with waiting lists). SSIs offer transdiagnostic solutions to support the resilience of older adults and their care partners given their brief, targeted nature and potential to yield significant benefits in less time. These approaches have the potential to disseminate new approaches to care delivery, re-allocate resources, promote patient-centered care, and support positive outcomes (89). SSIs can be implemented as stand-alone services or as adjunctive care to improve treatment engagement and can be tailored to multiple points in care journeys. SSIs can also be used to facilitate adjustment to care transitions. For healthcare systems and communities, intervention development and implementation frameworks should be consulted to evaluate what structure and supports are needed to encourage the development of SSIs in high-need settings, with specific attention to the considerations of older adults (89).
